# 

**Published:** 1897-04-17

**Authors:** 


### EARLY EDITION.

**Notices of Births, Deaths, and Marriages** are inserted in this column at a charge of 2s. 6d. for an announcement not exceeding four lines.

### NOTICE TO CORRESPONDENTS.

All MS., Letters, Books for Review, and other matters intended for the Editor should be addressed The Editor, "The Hospital," The Hospital Building, 28 & 29, Southampton Street, Strand, W.C.

The Editor cannot undertake to return rejected MS., even when accompanied by stamped directed envelope.

**Notice to Advertisers and Subscribers.**

All Advertisements, Orders for Copies of The Hospital, and Business Communications should be addressed to **THE MANAGER, (Not to The Editor),** The Hospital, The Hospital Building, 28 & 29, Southampton Street, Strand, London, W.C.

The Cost of Subscription, post free, is as follows:—Per week, 2½d.; per quarter, 2s. 8d.; per half-year, 5s. 3d.; per annum, 10s. 6d. Foreign:—Per annum, 15s. 2d.; payable, in all cases, in advance.

**NOTICE.—Vol. XXI. of "The Hospital" (October, 1896, to March, 1897), in handsome cloth binding, is on sale at the Office of this Journal, price 7s. 6d. Cases for binding the Numbers contained in this Volume, 1s. 6d. Index to Vol. XXI., 2½d., post free.**

**The Monthly Part for May will be ready on May 1st. Price 6d., by post 8d.**

### SCRAPS AND GLEANINGS.

IT would appear from the reports which reach us that, nearly all over the kingdom, the chief form which the local celebration of the sixtieth anniversary of Her Majesty's reign is to take is a special effort to place the local hospital on a better financial footing.

MONSIEUR FELIX FAURE, the President of the French Republic, has, through the French Ambassador in London, replied to the hon. secretary of the Imperial Fête, that while most cordially sympathising with the object of the fête, he much regrets his inability to become a patron.

THIRTY-THREE patients were under treatment at the Coldstream Cottage Hospital during the year just ended, or 14 fewer than in the previous year. The falling off is accounted for by the time (3½ months) during which the hospital was closed for heating and improvements.

SOME of the trading companies of Belfast are giving in a most liberal spirit towards the New Victoria Hospital, Belfast; one of them, the York Street Flax Spinning Company, Limited, having recently promised a donation of £5,000. Over one half of the £100,000 asked for has been received.

